# A prospective study of the muscle strength and reaction time of the quadriceps, hamstring, and gastrocnemius muscles in patients with plantar fasciitis

**DOI:** 10.1186/s12891-020-03740-1

**Published:** 2020-11-05

**Authors:** Jin Hyuck Lee, Hae Woon Jung, Woo Young Jang

**Affiliations:** 1 Department of Sports Medical Center, Korea University College of Medicine, Anam Hospital, Seoul, Republic of Korea; 2 Department of Pediatrics, Kyung Hee University Medical Center, Seoul, South Korea; 3 Department of Orthopedic Surgery, College of Medicine, Korea University, 73, Inchon-ro, Seongbuk-gu, Seoul, 02841 Republic of Korea

**Keywords:** Plantar fasciitis, Muscle reaction time, Foot pressure, Pedobarography, Gastrocnemius

## Abstract

**Background:**

Muscle weakness is an important etiological factor in plantar fasciitis (PF), but available data on the role of the quadriceps, hamstring, and gastrocnemius (GCM) muscles are limited. The aim of this study was to compare the strength and reaction time of the quadriceps, hamstring, and GCM muscles and foot pressure between patients with PF and normal controls.

**Methods:**

A total of 21 PF patients and 21 normal controls were enrolled. Muscle strength was measured by the peak torque per body weight (Nmkg^− 1^ × 100). Muscle reaction time was evaluated by the acceleration time (AT, milliseconds). Foot pressure and posture were assessed by pedobarography [valgus/varus index (VV index), %].

**Results:**

The strength of the quadriceps was significantly lower in the affected ankles of the PF group than in the control group (*p =* 0.005). The AT of the quadriceps and hamstring muscles was significantly increased in the affected ankles of the PF group than in the control group (quadriceps: *p =* 0.012, hamstring: *p =* 0.001), while the AT of the GCM muscle was significantly decreased (*p =* 0.009) and significantly correlated negatively with quadriceps muscle strength (r = −.598, *p* = 0.004) and AT (r = −.472, *p* = 0.031). Forefoot (*p =* 0.001) and hindfoot (*p =* 0.000) pressure were significantly greater, with the VV index showing hindfoot valgus, in the affected ankles in the PF group compared to the control group (*p =* 0.039).

**Conclusions:**

This study demonstrated weakness and delayed reaction time of the quadriceps and hamstring muscles, with a rapid reaction time of the GCM muscle, in patients with PF.

**Clinical relevance:**

Clinicians and therapists should assess the function of the quadriceps and hamstring muscles when planning the management of PF patients without muscle tightness.

## Background

Plantar fasciitis (PF) is one of the most common problems associated with foot pain. The causes of PF include excessive physical activity, [1] obesity, [2] age, [3] prolonged standing, [2] altered biomechanics, [4, 5] limited ankle dorsiflexion with foot postures such as pes cavus and pes planus, [6] and hamstring tightness [7]. Among these, limited ankle dorsiflexion is caused by tightness of the gastrocnemius (GCM), which may increase the stress on the plantar fascia because it affects the alignment of the calcaneal bones [8]. Hamstring tightness may induce prolonged forefoot loading, that can result in increased repetitive stress on the plantar fascia [7, 9]. Therefore, most therapists have focused on restoring the flexibility of the posterior muscles, such as the GCM and hamstring muscles in PF patients.

Weakness of the GCM [5] and proximal muscles, [5, 10] such as the gluteal and tensor fasciae latae muscles, in patients with PF have been reported, which may impact the plantar fascia load distribution. Recently, a systematic review reported that intrinsic muscle strength is associated with symptoms of PF [11]. Furthermore, a recent study by Lee et al., [5] reported that increased foot pressure in patients with PF may be associated with weakness of the GCM and hip muscles. Therefore, muscle weakness may be an important etiological factor in PF. To date, however, no study has investigated the strength and reaction time of proximal muscles, such as the hamstring and quadriceps muscles, in patients with PF. These muscles are known to play a vital role in the alteration of lower extremity biomechanics [12–15] and may contribute to increased plantar fascia load.

The purpose of this study was to analyze the differences in the strength and reaction time of the quadriceps, hamstring, and GCM muscles as well as foot pressure and posture, between patients with PF and normal controls. We hypothesized that the quadriceps, hamstring, and GCM muscles of PF patients would show decreased strength and delayed reaction time, and these patients would have increased foot pressure compared to normal controls.

## Methods

### Participants

**Fig. 1:**
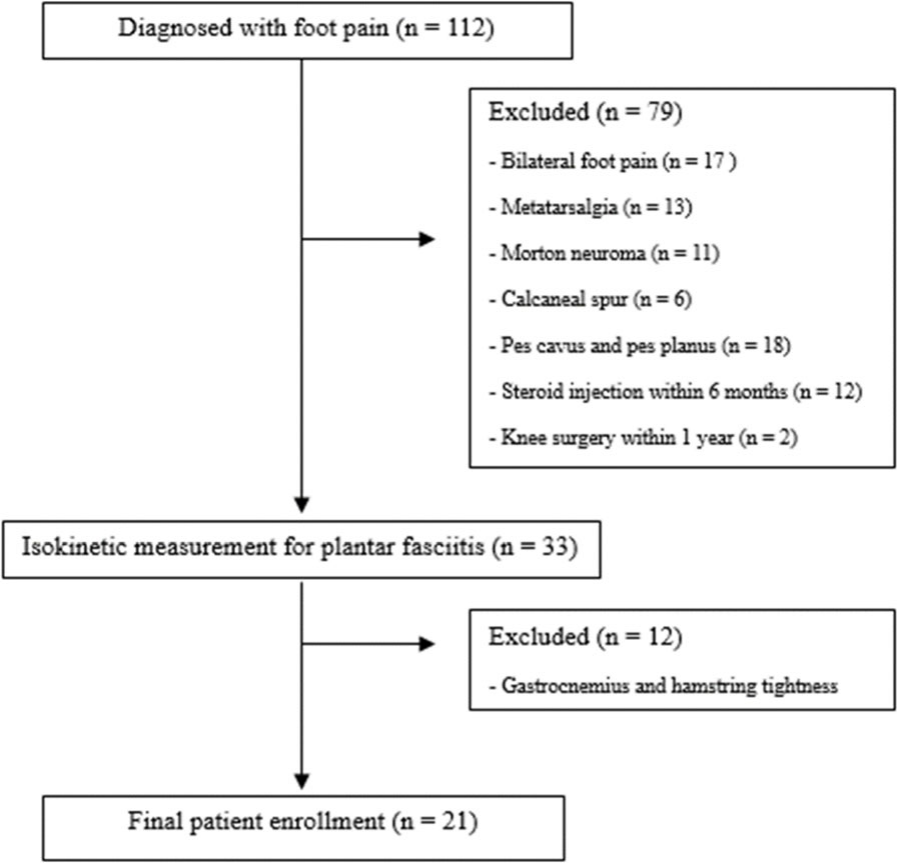
Flowchart of patients with plantar fasciitis

This study was approved by our institutional review board. All study participants provided written informed consent, and the rights of the subjects were protected. This study is a prospective, investigator-initiated trial. All data were managed in Excel files by a blinded author, and statistical analyses were done by a statistician. This prospective case-control study enrolled 112 patients with foot pain at our institute between July 2018 and November 2019. Physical examinations and evaluations of all images were independently performed by two experienced surgeons. Any disagreements on any diagnoses of PF were resolved by consensus. In this study, the inclusion criteria were PF patients with normal foot posture in terms of naviculocuboid overlap and talonavicular coverage angle on plain radiographs, without tightness of the GCM or hamstring muscles. We excluded 91 patients for the following reasons (Fig. 1): pain in both feet, metatarsalgia, Morton neuroma, calcaneal spur, pes cavus and pes planus, and tightness of the GCM and hamstring muscles in the Silfverskiold and popliteal angle tests, respectively. We also excluded patients who had received a steroid injection within 6 months or had undergone knee surgery within 1 year. Of the 112 patients, 91 were excluded; therefore, 21 patients were finally enrolled. The 21 normal control subjects selected from our database of volunteers had no history of lower extremity injury symptoms within 1 year and agreed to participate in the study.

### Assessment of isokinetic muscle performances

#### Muscle strength of the quadriceps and hamstring

**Fig. 2:**
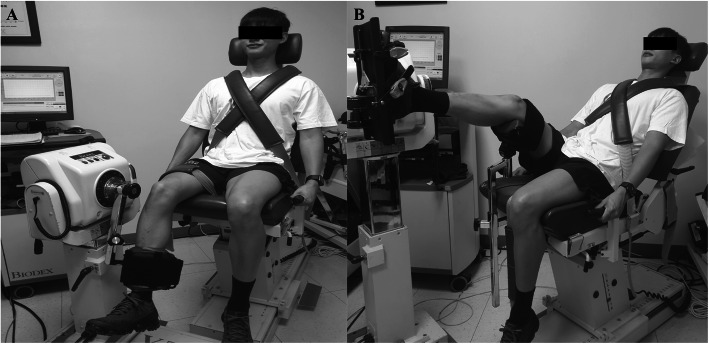
Measurement of the strength and reaction time of the quadriceps, hamstring (**a**), and gastrocnemius (**b**) muscles

Isokinetic knee extension/flexion strength (concentric/concentric mode, Nmkg^− 1^ × 100, Biodex Medical Systems, Shirley, NY) was measured in the sitting position with 90° flexion of the hips and knee joints on a dynamometer (Fig. 2a). Flexion and extension strength were considered to represent hamstring and quadriceps strength, respectively. Each test consisted of 5 repetitions of flexion/extension (ROM, 90° to 0°) for each leg at 60°/s.

#### Muscle strength of the GCM

Isokinetic GCM strength (concentric mode, Nmkg^− 1^ × 100) was measured in a semi-seated position with 20° of knee flexion [16] on a dynamometer (Fig. 2b), and 5 repetitions of plantar flexion for each leg at 30°/s.

#### Assessment of the muscle reaction time (acceleration time)

Muscle reaction time was measured by the acceleration time (AT) during isokinetic strength testing. Muscle reaction time was defined as the time (ms) required to attain the pre-set angular velocity (60°/s for the knee joint and 30°/s for the ankle joint) during maximal muscle contraction. Lower AT values signify a rapid muscle reaction ability [17–19]. The AT was calculated automatically using the Biodex advantage software.

#### Assessment of the foot pressure and posture

**Fig. 3:**
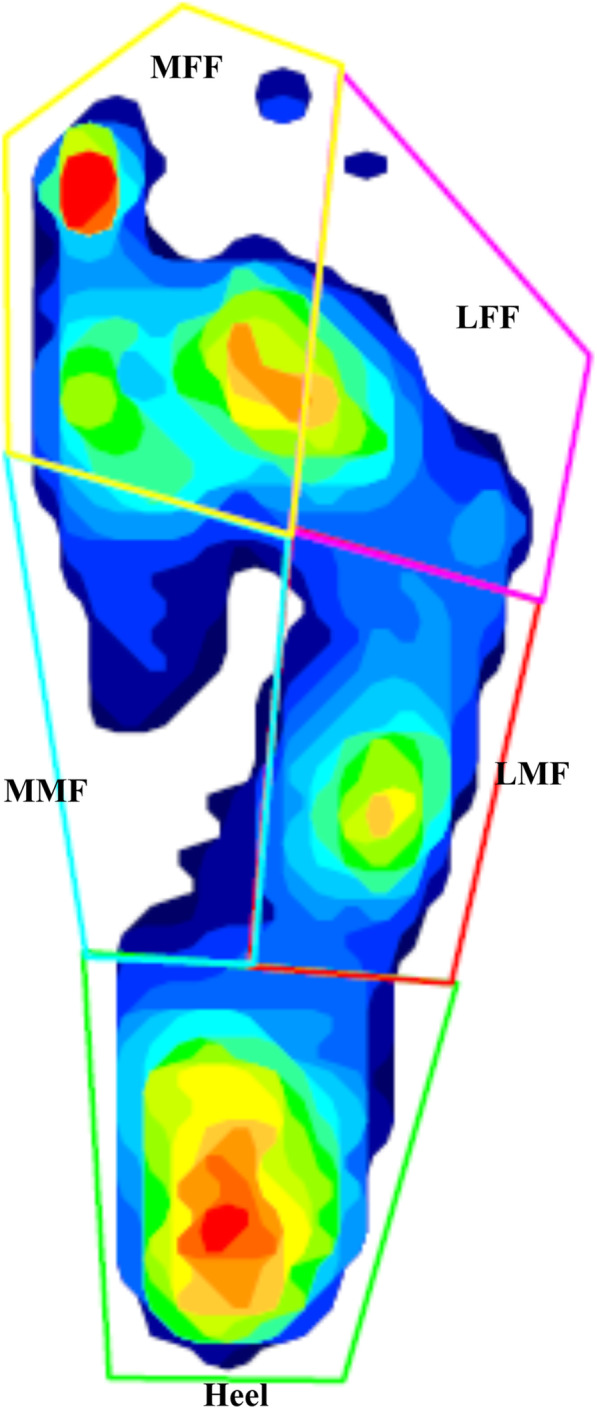
Five segments on pedobarography: the medial forefoot (MFF), lateral forefoot (LFF), medial midfoot (MMF), lateral midfoot (LMF), and heel. This image shows hindfoot valgus with increased pressure in the forefoot and hindfoot

Foot pressure was measured by pedobarography [5, 20, 21] (Tekscan, Massachusetts) during a 2-m walk and recorded at 50 Hz. Based on a previous study, [21] the peak pressure and pressure–time integral were calculated for each of the 5 segments of the foot (Fig. 3): the medial forefoot (MFF), lateral forefoot (LFF), medial midfoot (MMF), lateral midfoot (LMF), and heel. These data were processed to yield the valgus/varus index (VV index, %), which is defined as ((MMF + MFF) - (LMF + LFF))/(MMF + MFF + LFF + LMF), with plus (+) and minus (−) values of the VV index indicating hindfoot valgus and varus, respectively [21]. The same peak pressure and VV index assessment processes were used for the normal controls.

### Statistical analysis

The sample size calculation for this study was based on a previous study of muscle strength in patients with lower extremity injuries, [19, 22] and a muscle strength difference > 10% between the groups was considered significant. To determine the sample size, we conducted an a priori power analysis, with an alpha level of 0.05, and a power of 0.8. Effect size (Cohen’s d: 1.00) was calculated using the mean and standard deviation from the results of a pilot study involving 5 ankles in each group; 17 ankles in each group were required to adequately identify a clinically meaningful difference of > 10% in muscle strength between the groups. The power necessary to detect differences in muscle strength was 0.813.

The Student’s *t*-test was used to compare the strength and reaction time of the quadriceps, hamstring, and GCM muscles, and the foot pressure and posture between patients with PF and normal controls. To determine whether a continuous variable followed a normal distribution, the Shapiro test was used. Correlations between the strength and reaction time of the quadriceps, hamstring, and GCM muscles were assessed using Pearson’s coefficient of correlation. Data were analyzed using SPSS software version 17.0 (SPSS Inc., Chicago, IL, USA). A value of *p* < 0.05 was considered statistically significant.

## Results

Table 1 shows the demographic data of the PF patients and normal controls. There were no differences in sex, age, height, weight, or sports and activity levels [23] (high level was defined as participation in competitive sports such as basketball, volleyball, football, and soccer) between the two groups.

**Table 1:** Demographic data in enrolled patients with plantar fasciitis and normal controls

	PF patients group (*n* = 21)	Normal control group (n = 21)	*p*-value
Sex (male/female)	10/11	13/8	0.365
Age (years) ^a^	53 ± 4	51 ± 7	0.342
Height (cm) ^a^	168 ± 3	166 ± 6	0.697
Weight (kg) ^a^	66 ± 7	68 ± 4	0.778
Sports and activity, n (low:high)	18:3	16:5	0.401

*Abbreviations*: *PF* plantar fasciitis

^a^The values are expressed as mean ± standard deviation

### Isokinetic strength

The strength of the quadriceps, but not the hamstring or GCM (*p* > 0.05, Table 2), was significantly decreased in the affected ankles of patients with PF compared with those of normal controls (115 ± 34.7 vs. 144 ± 26.1, respectively; *p =* 0.005, Table 2).

**Table 2:** Comparison of muscle strength and acceleration time in both ankles between the patients with plantar fasciitis and normal controls

	Affected ankles			Unaffected ankles		
	PF patients group	Normal control group	*p*-value	PF patients group	Normal control group	*p*-value
GCM strength	30 ± 11.4	41 ± 14.4	0.278	37 ± 10.9	41 ± 11.5	0.633
Quadriceps strength	115 ± 34.7	144 ± 26.1	**0.005** ^**a**^	126 ± 34.8	141 ± 21.9	0.110
Hamstring strength	61 ± 20.4	68 ± 12.7	0.182	74 ± 16.9	77 ± 8.2	0.370
GCM AT	30 ± 11.4	41 ± 14.4	**0.009** ^**a**^	37 ± 10.9	41 ± 11.5	0.278
Quadriceps AT	64 ± 25.2	48 ± 14.4	**0.012** ^**a**^	54 ± 25.2	51 ± 14	0.652
Hamstring AT	77 ± 21.9	56 ± 15.6	**0.001** ^**a**^	60 ± 13.7	58 ± 17.7	0.629
Forefoot pressure	70 ± 27.7	46 ± 15.7	**0.001** ^**a**^	52 ± 18.7	46 ± 15.7	0.277
Hindfoot pressure	65 ± 22.8	36 ± 15.2	**0.000** ^**a**^	44 ± 18.6	36 ± 15.2	0.115
Foot posture (VV index)	0.2 ± 0.3	0 ± 0.2	**0.039** ^**a**^	−0.1 ± 0.3	0 ± 0.2	0.861

*Abbreviations*: *PF* plantar fasciitis, *GCM* gastrocnemius, *AT* acceleration time, *VV index* valgus/varus index

Note: The values are expressed as mean ± standard deviation

Measurement units for muscle strength and muscle reaction time were Nm kg^− 1^ × 100 and milliseconds, respectively

^a^Statistically significant

### Muscle reaction time (AT)

The AT of the hamstring and quadriceps muscles was significantly greater in the affected ankles of the PF group than in those of the control group (hamstring: 77 ± 21.9 vs. 56 ± 15.6, *p =* 0.001, quadriceps: 64 ± 25.2 vs. 48 ± 14.4, *p =* 0.012, Table 2), whereas the AT of the GCM muscle was significantly lower in the PF patients than in the normal controls (30 ± 11.4 vs. 41 ± 14.4, *p =* 0.009, Table 2).

### Correlations between the strength and reaction time of the quadriceps, hamstring, and GCM muscles

The strength of the GCM muscle in the affected ankles showed a significant positive correlation with the strength of the hamstring muscle (r = .634, *p* = .002, Table 3), but not with the quadriceps muscles (*p* > 0.05, Table 3). The AT of the GCM muscle in the affected ankles showed a significant negative correlation with the strength (r = −.598, *p* = .004, Table 3) and AT (r = −.472, *p* = .031, Table 3) of the quadriceps muscle, but not with the hamstring muscle (*p* > 0.05, Table 3).

**Table 3:** Correlations between the muscle strength and muscle reaction time

Parameters		Affected ankles		Unaffected ankles	
		GCM strength	GCM AT	GCM strength	GCM AT
Quadriceps strength	PCC (r)	.289	−.598	.277	− 252
	*p*-value	.204	**.004** ^**a**^	.225	.271
Hamstring strength	PCC (r)	.634	−.371	.632	−.113
	*p*-value	**.002** ^**a**^	.098	**.002** ^**a**^	.627
Quadriceps AT	PCC (r)	−.533	−.472	−.189	.080
	*p*-value	**.013** ^**a**^	**.031** ^**a**^	.412	.732
Hamstring AT	PCC (r)	−.357	.212	.213	−.351
	*p*-value	.112	.356	.354	.119

*Abbreviations*: *PCC* Pearson’s correlation coefficient, *GCM* gastrocnemius, *AT* acceleration time

^a^Statistically significant

### Foot pressure and posture (VV index)

Forefoot and hindfoot pressure were significantly greater in the affected ankles of patients with PF than in those of normal controls (forefoot: 70 ± 27.7 vs. 46 ± 15.7, *p =* 0.001, heel: 65 ± 22.8 vs. 36 ± 15.2, respectively; *p =* 0.000, Table 2). The VV index values revealed a higher incidence of hindfoot valgus in the affected ankles of patients with PF compared with those of normal controls (+ 0.2 ± 0.3 vs. 0 ± 0.2, respectively; *p =* 0.039, Table 2).

In the unaffected ankles, there were no significant differences in the strength and reaction time of the quadriceps, hamstring, and GCM muscles, nor the foot pressure and posture between the PF group and the control group (*p* > 0.05, Table 2).

## Discussion

The most important finding of this study was that quadriceps weakness, delayed reaction time of the hamstring and quadriceps muscles, and rapid reaction time of the GCM muscle can all be demonstrated in the affected ankles of PF patients. The reaction time of the GCM muscle also had a significant negative correlation with the strength and reaction time of the quadriceps muscle. Furthermore, foot pressure at the forefoot and hindfoot significantly increased, and the affected ankles of patients with PF had a higher incidence of hindfoot valgus than those of normal controls.

Weakness of the GCM in patients with PF has been reported [5, 24]. However, these studies investigated PF patients with concurrent tightness of the GCM muscle. Therefore, previous studies were limited because muscle length directly affects muscle strength [25]. However, In this study, PF patients without muscle tightness had weakness of the quadriceps muscle, with no significant difference in the strength of the hamstring and GCM muscles between the groups. Although the reason for these results is unclear, it may be explained by the use of compensatory movement strategies to reduce foot pain. During the gait cycle, [26] foot posture changes from supination to pronation during the change in phase from heel strike to weight acceptance. In patients with PF, foot pain may be due to a stretched plantar fascia in the pronated foot [26]. As a result, patients may use compensatory movement strategies, such as rapid hip flexion to reduce foot pain. In the weight acceptance phase, the quadriceps, hamstring, and GCM muscles, (especially the quadriceps), are highly active in stabilizing the hip and knee joints against gravity and weight [13, 14, 25]. However, in PF patients, the function of the quadriceps may be gradually reduced by insufficient weight transfer due to such compensatory strategies, thereby resulting in quadriceps muscle weakness. Another possible explanation is overuse of the hip flexion movement performed to reduce foot pain caused by a stretched plantar fascia. The quadriceps muscle is a hip flexor, and weakness in this muscle may result from its overuse [27, 28] in an effort to reduce foot pain. Previous studies have reported that decreased quadriceps strength can lead to increased plantar fascia load and decreased control of pronation of the foot, [26, 29] thereby increasing foot pain. Further prospective studies are necessary to elucidate the results of PF patients in this study.

In the PF patients in this study, the reaction time of the hamstring and quadriceps muscles was delayed, whereas the reaction time of the GCM muscle was rapid compared to those of the control group. We believe that these results may be attributable to joint stabilization strategies in the lower extremity. Muscle reaction can be defined as the ability of the muscle to maintain joint stability while performing a functional task [30, 31]; thus, rapid muscle reaction time is an important factor for increased joint stability [31, 32]. The quadriceps, hamstring, and GCM muscles all contribute to the stability of the knee joint. Lloyd and Buchanan reported that the co-contraction of the quadriceps and hamstring muscles directly supports the valgus and varus moments at the knee joint [33]. The valgus and varus moments of the knee joint can impact foot pronation and supination, respectively, [34–36] which may increase plantar fascia stress owing to increased pressure in the forefoot and the hindfoot [20]. In PF patients in this study, the hamstring and quadriceps muscles showed a delayed reaction time, with greater pressure in the forefoot and hindfoot, and hindfoot valgus on pedobarography, despite having normal feet on plain radiographs, compared to those in the control group. Hence, functional abnormalities of the hamstring and quadriceps muscles may contribute to increased pressure in the forefoot and hindfoot. In particular, the reaction time of the GCM muscle showed a significant negative correlation with the strength and reaction time of the quadriceps muscles in this study. Therefore, we believe that the GCM muscle may respond rapidly to support the valgus/varus moments in patients with PF whose hamstring and quadriceps muscles have a delayed reaction time. Previous studies have also reported that the GCM muscle plays an important role in supporting the frontal plane knee alignment (valgus/varus moments) at the knee joint [33, 37]. Kvist and Gillquist, [38] and Meunier et al. [39] reported that the GCM muscle is neurologically connected to the quadriceps muscle. Consequently, we believe that the reaction time of the hamstring and quadriceps muscles should be assessed and improved, as necessary, in patients with PF.

There were several limitations to the present study. First, the strength of gluteal and hip muscles, such as the hip abductors, was not evaluated in this study, even though previous studies [5, 10, 40] have reported that hip muscle strength is closely related to foot pain. Second, post rehabilitation results were not included in the correlation analysis. To confirm that the functional abnormalities of the hamstring and quadriceps muscles shown in our results represent a definite etiology of PF in patients without tightness of the GCM and hamstring muscles, further evaluations of the quadriceps and hamstring muscles should be done following rehabilitation. In addition, further studies on how the performance of the hamstrings, quadriceps, and GCM muscles of patients who were excluded from this study will contribute to PF will also improve our understanding of PF in various patients. Finally, intrinsic foot muscle function was not assessed. Intrinsic foot muscles play an important role in the stability of the normal foot and in lower extremity function; thus, impairment of these muscles may affect lower extremity biomechanics, which may result in changes in the function of the quadriceps, hamstring, and GCM muscles.

## Conclusions

This study revealed the presence of weakness and delayed reaction time of the hamstring and quadriceps muscles, and a rapid reaction time of the GCM muscle in patients with PF. Clinicians and therapists should aim to evaluate and improve the functionality of these muscles in patients with PF.

## Data Availability

The datasets used and/or analyzed in this study are available from the corresponding author upon reasonable request.

## Abbreviations

PF: Plantar fasciitis
GCM: Gastrocnemius
AT: Acceleration time
MFF: Medial forefoot
LFF: Lateral forefoot
MMF: Medial midfoot
LMF: Lateral midfoot
VV index: Valgus index
